# Neonatal breast-suckling skills in the context of lactation and peripartum hormonal changes and additional factors—a pilot study

**DOI:** 10.1186/s13006-022-00508-2

**Published:** 2022-09-01

**Authors:** Katarzyna Maria Wszołek, Karolina Chmaj-Wierzchowska, Małgorzata Pięt, Agata Tarka, Marek Chuchracki, Błażej Męczekalski, Maciej Wilczak

**Affiliations:** 1 Department of Maternal and Child Health, Poznan University of Medical Sciences, Poznań, Poland; 2 Gynecology-Obstetrics Clinical Hospital, Poznan University of Medical Sciences, Poznań, Poland; 3 Facility of Practical Midwifery Sciences, Poznan University of Medical Sciences, Poznań, Poland; 4 Faculty of Health Sciences, Chair of Cosmetology, Calisia University, Kalisz, Poland; 5 Department of Gynecological Endocrinology, Poznan University of Medical Sciences, Poznań, Poland

**Keywords:** Breastfeeding, Prolactin, Cortisol, Breast-suckling skills, Support

## Abstract

**Background:**

Childbirth and lactation are intricate processes, involving several hormones, the most important of which are prolactin (a protein hormone) and cortisol (one of the glucocorticoids). The early postpartum period is crucial for both mother and newborn and has an impact on the lactation and breastfeeding process.

**Methods:**

The study included 78 patients who were admitted to the Gynecology-Obstetrics Clinical Hospital in Poznań for labor induction and/or in the active phase of the first labor stage. The levels of cortisol and prolactin in serum were assessed in these women during admission in labor, during the third labor stage, and on the second day postpartum. The levels of cortisol and prolactin in the umbilical cord serum were assessed immediately after cord clamping. The “Protocol for the assessment of breast-suckling skills” was used to assess the neonatal breast-suckling skills on the second day postpartum. Some additional parameters were evaluated in mothers via a telephone interview at three and six months postpartum. The study was conducted from January to August 2020, however the study was suspended during April–July 2020 due to the SARS-CoV-2 pandemic, which led to restrictions in the hospital limiting access to the hospital wards unless necessary.

**Results:**

Early breastfeeding with skin-to-skin contact was associated with low levels of hormones, cortisol levels were lower in serum (*p* = 0.0108) and umbilical vein (*p* = 0.0273) in mothers who breastfed immediately after childbirth. At three months postpartum, 88% of the mothers who did not offer a pacifier to the child during the first few days of life breastfed the child naturally (*p* = 0.037), and at six months, 96% of those who did not offer a pacifier continued to breastfeed (*p* = 0.0008). Multiple, statistically significant correlations were observed between the variables assessed according to the “Protocol for the assessment of breast-suckling skills” and breastfeeding after three months.

**Conclusions:**

Breastfeeding immediately after childbirth, appropriate assessment of the breast-suckling skills of newborns, avoiding pacifiers and infant formula feeding, and offering support to new mothers in the early days after childbirth seem to be important factors for sustaining breastfeeding after three and six months of childbirth.

## Background

Childbirth and lactation are intricate processes, involving several hormones including prolactin, oxytocin, cortisol, estrogen, and progesterone [1, 2]. The precise determination of the interactions occurring between these hormones and assessment of changes in their concentrations during the perinatal period is challenging because at this time complex mechanisms take place in the body of both the mother and the fetus [2].

The role of prolactin in the maternal and neonatal body is associated with many physiological processes, such as conception, postpartum maternal adaptations (including personality changes), lactation, and infant growth and brain development [3]. Prolactin is a protein hormone which belongs to the prolactin/growth hormone/placental lactogen family and is synthesized and released by the cells of the anterior pituitary gland [4]. The secretion of this hormone is promoted by estrogen, thyroid-releasing hormone, norepinephrine, oxytocin, and some forms of stress; however, the release of prolactin is triggered mainly during the childbirth and lactation process [3]. The success of breastfeeding, which is understood as producing sufficient milk to meet the newborn’s nutritional and immune requirements, is influenced by several factors [5–7]. Cortisol is one of the glucocorticoids synthesized by the adrenal cortex and secreted by the hypothalamic hormone (corticotropin-releasing hormone) and pituitary hormone (adrenocorticotropic hormone) in the hypothalamic–pituitary–adrenal (HPA) axis. Cortisol is well-known as a stress hormone but, as a matter of fact, its impact on the maternal and fetal body during pregnancy and childbirth is wide and complex. It plays a role in the maturation of fetal lung and other organs, elevates prepartum fetal arterial pressure, and maintains maternal and fetal glucose levels during parturition [8, 9]. Early postpartum period is crucial for both the mother and the newborn. The hypersensitivity of their systems during this period is related to their requirement to stay together, with their bodies close, in a warm, calm, and dark environment. It is proven that disruption of mother–newborn interaction may have long-term consequences and influence the epigenetic changes since the neonatal hypothalamic hormone system is immature and vulnerable [10]. The available reports on the adverse effects of synthetic oxytocin on breastfeeding are conflicting. Studies have shown that women administered with synthetic oxytocin for inducing labor or enhancing contraction during labor breastfed their babies exclusively for a shorter time [9–15]. At the same time, in women who received an infusion of synthetic oxytocin during labor, significantly higher levels of prolactin were observed on the second day postpartum, as well as a decrease in endogenous oxytocin, particularly in those who received epidural analgesia during labor [14, 15]. The maternal cortisol plasma levels are elevated during childbirth, which represents hormonal adaptation to acute stress preventing maternal hypoglycemia [3, 9, 16]. On the other hand, breastfeeding seems to inhibit the extended increase in maternal cortisol levels [17, 18]. In Poland, the oral cavity structure of a newborn and his/her competence to suckle effectively are examined using the “Protocol for the assessment of breast-suckling skills,” developed by international lactation consultants in collaboration with pediatricians and neurologists [19]. The parameters mentioned in the protocol are assessed during the early postnatal days. The protocol is applicable for infants born between 37 and 42 weeks of fetal age. The first 12 h of a newborn’s life is the recommended time for the evaluation of the oral structure and oral reflex reactions according to the protocol. Grasping and suckling of the breast as well as the efficiency of food intake is assessed from the second day postpartum until discharge [19].

### Aim of the study

The study aimed to assess neonatal breast-suckling skills in the context of lactation and peripartum hormonal changes and additional factors.

## Methods

**Fig. 1:**
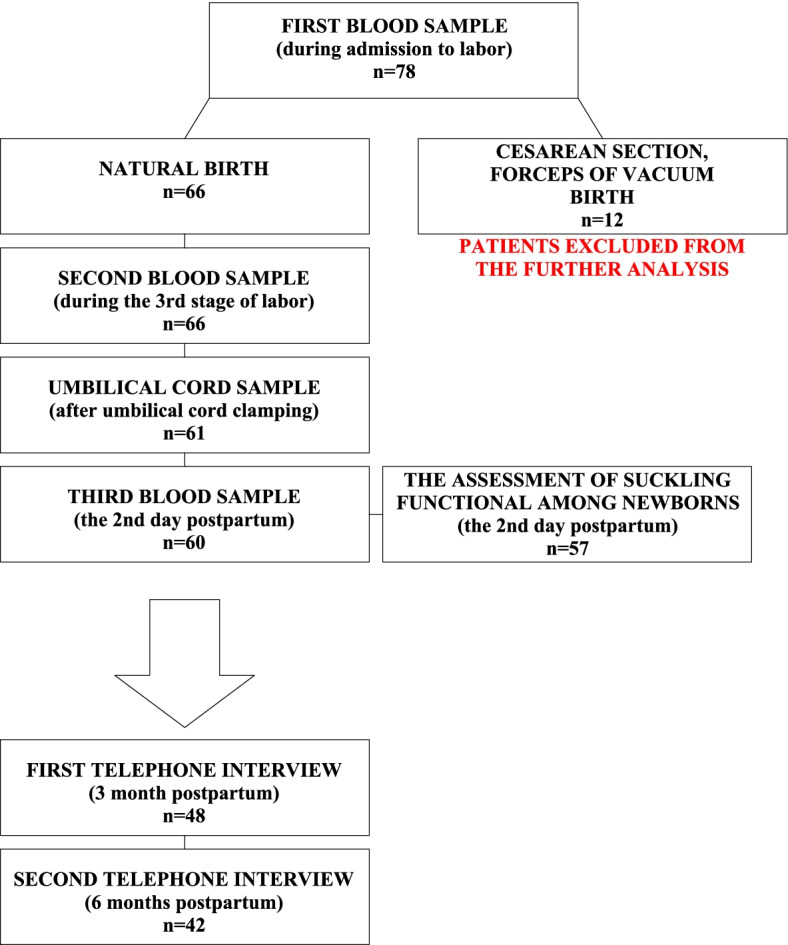
Schematic representation of the selection of patients

The study included a total of 78 full-term pregnant women who were admitted to the Gynecology-Obstetrics Clinical Hospital in Poznan for labor induction and/or in the active phase of the first labor stage, from January to August 2020, however the study was suspended during April–July 2020 due to the SARS-CoV-2 pandemic, which led to restrictions in the hospital limiting access to the hospital wards unless necessary. The inclusion criteria for the study were as follows: single pregnancy, no contraindications to natural delivery at the time of qualification, gestational age between 37 and 42 weeks, and no fetal anomalies. Written informed consent was obtained from all participants. The levels of cortisol and prolactin in serum were assessed in the participants during admission to labor, during the third stage of labor and before infusion with oxytocin in women who gave birth naturally, and on the second day postpartum. The cortisol and prolactin levels in umbilical cord serum were assessed immediately after cord clamping (Fig. 1).

Blood was collected from a venous vessel (or umbilical vein) using a closed aspiration and vacuum set SARSTEDT S-MONOVETTE 9 mL, containing a clotting activator (silicate). The collected sample was labeled with the date and time of collection and transferred to the laboratory for the analysis. The evaluation of cortisol and prolactin levels was performed by electrochemiluminescence using Cobas 6000 apparatus. Considering the daily fluctuations in serum cortisol levels, the first sampling was performed in the morning. Before the cortisol analysis, the hourly range, corresponding to the hours of sampling, was marked in relation to the cortisol test (6–10 and 16–20). In the case of high hormonal concentrations, reassessment was performed after sample dilution.

In the second part of the study, the suckling function of naturally born newborns was assessed on the second day postpartum using selected elements of the “Protocol for the assessment of breast-suckling skills.” The assessment was carried out by two blinded midwives, an International Board of Certified Lactation Consultant (IBCLC) and a Certified Lactation Educator (CLE), who were not informed of induction, augmentation, or active management of labor with synthetic oxytocin.

The applied protocol assesses individual anatomical elements (lips, cheeks, mandible, tongue, hard palate, and tongue frenulum) and reflexes (searching, suckling, and biting) in newborns. For each of the listed parameters, 1 point is awarded for normal behaviors and 0 points for abnormalities. A total of 9 points indicates normal oral structure and reflexes, while 0–8 points indicate abnormalities in the analyzed parameters. After the assessment of oral structure and reflexes, suckling and grasping is assessed. Newborns who score 10 points for suckling and grasping are assessed further for milk intake efficiency. For newborns who score 8–9 points, correction of the way of breast suckling or grasping is indicated, while for those with 0–7 points consultation with a lactation consultant or neurologist is recommended. Regarding food intake efficiency, a score of 6 points indicates efficient milk intake by the child and the child can be assessed further for daily feeding efficiency, whereas if the child shows ineffective milk intake characteristics (0–5 points) the mother should consult a lactation consultant.

In the next stage of the study, each participant was contacted via telephone at three and six months postpartum to obtain information on the continuation of breastfeeding, the number of feeds per day, the sleeping pattern and current body weight of the child, the use of pacifiers, and the need to undercut the sublingual frenulum, as well as to understand the mother’s assessment of the child’s behavior.

The reasons for the loss of patients during the study were early discharge with the newborn to home (6 patients) and hospitalization of the newborn in the neonatal unit (1–3 children at different time points). Finally, the suckling function was assessed in 57 newborns. After 3 months, 48 patients responded when contacted by phone, and after six months, 42 patients responded.

The normality of the distribution of the tested variables was checked using the Shapiro–Wilk test. The results were presented as arithmetic mean and standard deviation for variables with normal distribution, and as median and the largest (maximum) and smallest (minimum) values in the case of variables with nonnormal distribution. The statistical significance of the studied dependencies and differences was assessed at *α* = 0.05. For quantitative variables with normality, parametric tests namely *t*-Student and ANOVA were used, while for variables with nonnormal distribution and for variables on an ordinal scale, nonparametric tests namely Mann–Whitney, Spearman rank correlation coefficient, and Kruskal–Wallis test were used. Nominal-scale variables were analyzed using the Fisher–Freeman–Halton test. Data analysis was performed using Dell Statistica (data analysis software system, version 13, software.dell.com) and Cytel Studio v.11.1.0.

## Results

### Characteristics of the study group

The age of the study group ranged from 21 to 41 years. Among the included women, 45 were multiparous, while 21 were primiparous. Twenty-four women had a natural birth without incision (36%), whereas 42 gave birth naturally with incision (64%). A total of 25 (38%) female and 37 (56%) male children were born. No data on sex were available for four (6%) babies. Among the studied women, 14 (21%) had gestational diabetes G1 and G2, 25 (38%) had hypothyroidism, and 18 (27%) had other diseases.

### Course of labor and puerperium

The duration of the first labor stage varied widely, ranging from 40 min to 17 h 30 min. The condition of the neonates, as assessed using the Apgar scale, was good or average. At 1 min of life, 56 (90.3%) neonates scored 10 points on the scale, while four (6.5%) scored 9 and one neonate each (1.5%, respectively) scored 8 and 6 points. At 5 min of life, only one neonate (1.5%) scored 9 points, while the remaining 61 (98.5%) scored 10 points. Among the patients who gave birth naturally, labor was induced in 14 (21%). In seven parturient women (11%), an intravenous infusion of oxytocin was administered in the first labor stage to enhance contractile function. In 12 parturient women (18%), oxytocin was administered in the second labor stage to enhance systolic function. In 39 women (59%), oxytocin was used for the active management of labor after cutting the umbilical cord of the neonate. Forty-five women (68%) breastfed their babies in the first two hours after delivery.

### Prolactin and cortisol correlations

Early breastfeeding with skin-to-skin contact had a significant impact on the maternal and umbilical serum cortisol levels, as cortisol levels were lower in serum (*p* = 0.0108) and umbilical vein (*p* = 0.0273) in mothers who breastfed immediately after childbirth. More interestingly, in mothers who breastfed the newborn with skin-to-skin contact, the serum prolactin concentration before childbirth was statistically significantly higher compared to mothers who did not breastfeed the baby at this time (*p* = 0.0099) and the serum cortisol concentration significantly decreased after childbirth (*p* = 0.0105, Table 1).

**Table 1:** Maternal and umbilical cord vein prolactin and cortisol serum levels depending on early breastfeeding of the newborn baby

	**Early breastfeeding with skin-to-skin contact**		***P*-value**
**Maternal prolactin serum level before childbirth**	Yes (*n* = 46)	No (*n* = 14)	**0.0099**
	Me = 227.65 ng/mL	Me = 146.10 ng/mL	
**Maternal cortisol serum level before childbirth**	Yes (*n* = 46)	No (*n* = 14)	**0.0105**
	Me = 1097.00 ng/mL	Me = 1693.50 ng/mL	
**Maternal prolactin serum level immediately after childbirth**	Yes (*n* = 45)	No (*n* = 14)	0.4175
	Me = 223.70 ng/mL	Me = 199.80 ng/mL	
**Maternal cortisol serum level immediately after childbirth**	Yes (*n* = 45)	No (*n* = 14)	**0.0108**
	Me = 1808.00 ng/mL	Me = 2236.50 ng/mL	
**Umbilical cord prolactin serum level**	Yes (*n* = 44)	No (*n* = 14)	0.1644
	Me = 416.45 ng/mL	Me = 371.40 ng/mL	
**Umbilical cord cortisol serum level**	Yes (*n* = 44)	No (*n* = 14)	**0.0273**
	Me = 199.15 ng/mL	Me = 346.60 ng/mL	
**Maternal prolactin serum level on second day postpartum**	Yes (*n* = 45)	No (*n* = 14)	0.1254
	Me = 357.90 ng/mL	Me = 313.85 ng/mL	
**Maternal cortisol serum level on second day postpartum**	Yes (*n* = 45)	No (*n* = 14)	0.9361
	Me = 620.40 ng/mL	Me = 586.70 ng/mL	

### Assessing the neonatal suckling skills

The oral cavity structure and oral reflex responses were assessed according to the second section of the “Protocol for the assessment of breast-suckling skills,” and the results are shown in Table 2.

**Table 2:** Evaluation of oral cavity structure and oral reflex responses in a group of 57 newborns

	**Normal (1 point)**	**Abnormal (0 points)**	**No data**
Lips	56 (98%)		1 (2%)
Cheeks	56 (98%)		1 (2%)
Mandible	56 (98%)		1 (2%)
Tongue	55 (96%)	1 (2%)	1 (2%)
Hard Palate	57 (100%)		
Tongue Frenulum	52 (91%)	5 (9%)	
Seeking Reflex	56 (98%)	1 (2%)	
Suckling Reflex	56 (98%)	1 (2%)	
Biting Reflex	47 (82%)	10 (18%)	

The results of the assessment of breast grasping and suckling are shown in Table 3.

**Table 3:** Assessment of breast grasping and suckling in a group of 57 newborns

	**Normal (1 point)**	**Abnormal (0 points)**	**No data**
Opening the mouth, tongue position	49 (86%)	5 (9%)	3 (5%)
Angle between the lips	46 (81%)	8 (14%)	3 (5%)
Lips	47 (83%)	7 (12%)	3 (5%)
Nose and chin	51 (90%)	3 (5%)	3 (5%)
Cheeks	50 (88%)	2 (3%)	5 (9%)
Depth of grasp	45 (79%)	9 (16%)	3 (5%)
Position of the areola	50 (88%)	4 (7%)	3 (5%)
Champing or smacking	52 (92%)	2 (3%)	3 (5%)
Mother’s perceptions	47 (83%)	7 (12%)	3 (5%)
Nipple shape	48 (84%)	8 (14%)	1 (2%)

The food intake efficiency after breast grasping was assessed based on the third section of the “Protocol for the assessment of breast-suckling skills,” and the results are shown in Table 4.

**Table 4:** Evaluation of food intake efficiency in a group of 60 newborns

	**Normal (1 point)**	**Abnormal (0 points)**	**Not evaluated**
Suckling movements (before milk outflow)	59 (98%)	1 (2%)	-
Suckling movements (during outflow)	58 (97%)	2 (3%)	-
Suckling series	56 (93%)	3 (5%)	1 (2%)
Suckling rhythm	56 (93%)	3 (5%)	1 (2%)
Swallowing	54 (90%)	4 (7%)	2 (3%)
Length of feeding from one breast	56 (93%)	2 (3%)	2 (3%)

The mothers were contacted via telephone to obtain information on the following: child’s general behavior, sleeping through the night, use of a pacifier, way of feeding, number of feedings per 24 h, and the need to undercut the tongue frenulum (Table 5).

**Table 5:** Parameters assessed in a telephone interview with the mother at 3 and 6 months postpartum

	**After 3 months (*n* = 48)**	**After 6 months (*n* = 42)**
**Child behavior**	Energetic, *n* = 10 (21%)	Energetic, *n* = 11 (26%)
	Crying/nervous, *n* = 6 (12.5%)	Cheerful, *n* = 10 (24%)
	Cheerful, *n* = 13 (27%)	Calm, *n* = 21 (50%)
	Calm, *n* = 19 (39.5%)	
**Sleeping through the night**	Yes, *n* = 15 (31%)	Yes, *n* = 13 (31%)
	No, *n* = 33 (69%)	No, *n* = 29 (69%)
**Using a pacifier**	Yes, *n* = 29 (60%)	Yes, *n* = 25 (60%)
	No, *n* = 19 (40%)	No, *n* = 17 (40%)
**Feeding**	Exclusively, *n* = 33 (69%)	Exclusively, *n* = 28 (67%)
	Mixed, *n* = 10 (21%)	Mixed, *n* = 5 (12%)
	Artificial, *n* = 5 (10%)	Artificial, *n* = 9 (21%)
**Number of feedings per day**	Me = 10	Me = 7
	Min. 5	Min. 1
	Max. 15	Max. 12
**Tongue frenulum undercutting**	Yes, *n* = 6 (13%)	Yes, *n* = 1 (2%)
	No, *n* = 41 (85%)	No, *n* = 41 (98%)
	Planned, *n* = 1 (2%)	

On the second day of life, 10 newborns (18%) received infant formula, while 50 did not (82%). The fact of formula feeding at such an early age was found to have a significant influence on how the infant was fed three and six months after birth. Of the 30 women who were still breastfeeding at three months, 91% did not provide formula to infants during the hospital stay (*p* = 0.042), whereas out of 28 women who were still breastfeeding at six months, only one fed formula to the infant at two days of life (*p* = 0.0375).

The parameters assessed on postnatal day two in relation to the way of feeding the baby at three months of age, according to the “Protocol for the assessment of breast-suckling skills,” are presented in Table 6.

**Table 6:** Correlation between the parameters assessed on second day postpartum and the way of feeding 3 months after childbirth

Assessed element	Way of feeding after 3 months	*P*-value
Tongue positioning	60% of children who had normally positioned tongue were fed naturally	**0.0397**
Searching reflex	57% of children who correctly expressed search reflex were fed naturally	**0.0410**
Suckling reflex	57% of children who showed normal suckling reflex were fed naturally	**0.0410**
Wide mouth opening	94% of naturally fed infants opened their mouths wide after the area below the nose was touched with the nipple during the assessment of suckling function	**0.0107**
Angle between the lips	88% of infants with an obtuse angle between the lips were fed naturally	**0.0267**
Depth of nipple grasp	85% of children who held a large part of the areola in the mouth were fed naturally	**0.0430**
Position of the areola	97% of children who correctly grasped the areola, with the lower lip covering more than the upper lip, were fed naturally	**0.0053**
Champing and smacking	None of the still naturally fed infants showed champing and smacking on day 2 of life	**0.0390**
Suckling movements assessed before feeding	Normal and fast movements were observed in all naturally fed infants	**0.0390**
Deep suckling movements	Normal deep suckling movements during milk outflow were observed in all children fed naturally	**0.0380**

The parameter assessed on postnatal day two in relation to the way the baby was fed at six months of age, according to the “Protocol for the assessment of breast-suckling skills,” was a wide mouth opening (all naturally fed infants opened their mouths wide during the assessment of suckling function, *p* = 0.015).

Among the analyzed women, 11 (19%) offered a pacifier to the baby on postnatal day two. At three months, 88% of the mothers who did not offer a pacifier breastfed the child naturally (*p* = 0.037), and at six months, 96% of those who did not offer a pacifier during the first few days of life continued to breastfeed (*p* = 0.0008).

## Discussion

Nipple stimulation and its timing during the breastfeeding process have a strong impact on the HPA axis, which causes a significant decrease in cortisol [18] and an increase in prolactin in plasma [20]. Our study showed the influence of early breastfeeding with skin-to-skin contact on maternal and umbilical cord serum cortisol levels which decreased if breastfeeding occurred early after childbirth (*p* = 0.0272).

The assessment of suckling function in a newborn during the first days of life and correction of abnormalities are extremely important for successful breastfeeding in later months. However, in order to breastfeed, which is a natural source of food, the neonate should exhibit certain skills, have properly developed oral cavity structures, and express appropriate oral reflexes [19]. Moreover, the effect of nipple shields used to protect the nipples on breastfeeding duration is unclear. Although nipple shields appear to have no impact on the plasma prolactin and cortisol levels among lactating mothers, they may reduce milk removal from the mammary gland [20–22]. In this study, the newborns’ suckling skills were assessed to determine the relationship between suckling skills and the duration of breastfeeding as well as the measured prolactin and cortisol levels. All the assessed newborns had a properly built hard palate (a parameter related to the structure of the oral cavity). The most frequently observed oral reflex abnormality (10 children, 18%) was an exaggerated biting reflex; however, it had no influence on further breastfeeding in the studied group. The basic reflexes of seeking and suckling were found to be important to maintain breastfeeding at three months of life, as well as the parameters related to breast grasping and suckling, namely champing and smacking, the depth of grasping the nipple and the correct embracing of nipple by the lips, the wide opening of the mouth before grasping the nipple, and the open angle between the lips after grasping the nipple. For efficient milk intake, the correct rhythm of suckling, series of suctions, and position of the tongue (an element of the oral cavity) were found to be crucial. For continuing breastfeeding at six months of life, the key parameter was the wide opening of the mouth after the baby’s medial cleft touches the nipple.

All subjects began breastfeeding in the hospital. After three months of childbirth, 52% of mothers were still exclusively breastfeeding, with the number of feedings per day varying between 5 and 15. After six months, 44% continued to breastfeed, with 1–12 feedings (on the child’s demand); all mothers had already introduced complementary feeding at this time and were not exclusively breastfeeding. The European Society for Paediatric, Gastroenterology, Hepatology and Nutrition (ESPGHAN) [23, 24] recommends baby-led weaning, which refers to introducing the baby to the taste of food, from the 17th week of life. However, this consists in an infant’s gradual learning of the taste of new products by licking and sucking, which does not replace breastfeeding and is therefore not qualified as full complementary feeding [24].

According to the report published by the Centre for Disease Control and Prevention (CDC) in 2020, in the United States 84.1% of mothers initiated exclusive breastfeeding in the hospital, and after three months, 46.9% were exclusively breastfeeding and at six months 25.6% continued exclusive breastfeeding [25]. These data and the results of the present study seem to be of concern because exclusive breastfeeding of infants up to six months of age is still the recommended mode of nutrition for the general population [23]. Complementary feeding is recommended only when an infant needs to be supplemented with foods rich in particular nutrients (e.g. iron) [25]. However, the expert panel of the Polish Society For Paediatrics Gastroenterology, Hepatology and Nutrition emphasizes that even partial or shorter breastfeeding can provide sufficient benefits to a child [23].

In this study, during telephone interviews at 3 and 6 months after birth, the reasons stated by mothers who had started regular feeding with modified milk or weaned the child from breastfeeding were milk shortage, lack of weight gain in the child, and frequent waking up and anxiety of the child (respondents believed that infants fed with modified milk were calmer). When asked about the help they received in terms of lactation counseling, most of the mothers indicated difficulties or, for various reasons, inability to obtain reliable advice, as well as a lack of knowledge about breastfeeding among midwives and pediatricians. The first two reasons indicate the need for structured lactation counseling after the discharge of mother and child from the hospital, with a focus on assessing the infant’s suckling function, observing the act of feeding, and evaluating the third stage of lactogenesis in mothers. These data are in line with a report published by the Centre for Lactation Science in 2018 [23], which highlighted that nearly 54% of Polish mothers who were discharged from the hospital felt that they would need additional assistance with breastfeeding. Moreover, the CDC experts [25] emphasize that mothers should be provided with individualized support in the first hours and days after birth to enable them to achieve their lactation goals.

The breastfeeding rates in Europe have been shown to be very low. Theurich et al. assessed breastfeeding rates in 11 European countries (Belgium, Croatia, Denmark, Germany, Ireland, Italy, The Netherlands, Norway, Spain, Sweden, and Switzerland) [26] and found that at the age of six months 35–65% of infants were breastfed and 13–39% were fully/exclusively breastfed in those countries. The relatively high rate of exclusive breastfeeding after 3 and 6 months postpartum (69% and 67%, respectively) observed in the present study may be related to the mothers’ decision to exclusively breastfeed their children for a long period and the breastfeeding consultation provided to them on the second day postpartum, of the two blinded midwives who assessed the neonatal suckling skills, one was an IBCLC and the other was a CLE.

In the context of the above recommendations and significant difference in breastfeeding rates between Europe and the United States, it seems reasonable to profoundly investigate the key reasons for difficulties in maintaining exclusive breastfeeding and develop ways to overcome them. Our study assessed term, healthy, naturally born babies, and even in our study group 14 (23%) mothers did not start breastfeeding during the first two hours postpartum. Moreover, 10 newborn babies assessed in our study (18%) were fed formula on the second day postpartum, which is consistent with the CDC report [25] showing that in 2017, infant formula was given to 19.2% of newborns even before the second day of life. Thus, a fundamental question arises regarding the prospect of the breastfeeding support offered in maternity wards and the multifactorial impact on the success of breastfeeding among new mothers [27, 28].

## Conclusions

Early breastfeeding after childbirth was associated with low serum cortisol and prolactin levels. In addition, the assessment of newborn’s suckling function during the first days of life and correction of abnormalities are important for successful breastfeeding in later months. It is necessary to implement interventions based on evidence-based medicine and evidence-based midwifery practice and offer reliable support to new mothers as soon as possible. Early implemented and personalized interventions as well as avoiding the use of a pacifier and formula feeds can contribute to maintaining breastfeeding for a long period.

## Data Availability

The datasets used and/or analyzed in the study are available from the corresponding author on reasonable request.
